# Lysozyme Aptamer-Functionalized Magnetic Nanoparticles for the Purification of Lysozyme from Chicken Egg White

**DOI:** 10.3390/foods8020067

**Published:** 2019-02-12

**Authors:** Ruiping Luo, Xinrui Zhou, Yan Chen, Sicheng Tuo, Fulin Jiang, Xiaodi Niu, Fengguang Pan, Hongsu Wang

**Affiliations:** College of Food Science and Engineering, Jilin University, Changchun 130062, China; luorp16@mails.jlu.edu.cn (R.L.); zhouxr14@mails.jlu.edu.cn (X.Z.); chen_y@jlu.edu.cn (Y.C.); tuosc9916@mails.jlu.edu.cn (S.T.); jiangfl9916@mails.jlu.edu.cn (F.J.); niuxd@jlu.edu.cn (X.N.)

**Keywords:** Fe_3_O_4_ nanoparticle, aptamers, lysozyme, immobilization, separation

## Abstract

Lysozyme is in high demand due to its many favorable characteristics such as being naturally occurring, non-toxic, and easy to digest and absorb. Recently, superparamagnetic nanoparticles with strong magnetic responsiveness have attracted significant interest for enzyme purification. The aptamer of the enzyme can be chemically synthesized rapidly at a large scale using simple and low-cost preparation methods. Therefore, Fe_3_O_4_ nanoparticles (Fe_3_O_4_ NPs) were prepared by chemical co-precipitation and were then functionalized with amino groups to produce NH_2_-Fe_3_O_4_ NPs. The specific reaction of aldehyde and amino groups was used to attach lysozyme aptamers with specific sequences to NH_2_-Fe_3_O_4_ NPs to produce Apt-NH_2_-Fe_3_O_4_ NPs. The synthesized materials were characterized using transmission electron microscopy (TEM), X-ray diffraction (XRD), Fourier transform infrared spectroscopy (FTIR), hysteresis loop analysis, and thermogravimetric analysis (TGA). The optimal experimental conditions for adsorption of lysozyme were investigated. The effects of initial lysozyme concentration, adsorption time, pH, reaction temperature, and ionic strength were determined. The maximum adsorption capacity and relevant activity of Apt-NH_2_-Fe_3_O_4_ NPs was 460 mg·g^−1^ and 16,412 ± 55 U·mg^−1^ in an aqueous lysozyme solution. In addition, as demonstrated by sodium dodecyl sulfate-polyacrylamide gel electrophoresis (SDS-PAGE) electrophoresis analysis, lysozyme could be separated from crude fresh egg white using Apt-NH_2_-Fe_3_O_4_ NPs with an amount up to 113 ± 4.2 mg·g^−1^ and an activity up to 16,370 ± 46 U·mg^−1^.

## 1. Introduction

Lysozyme is an alkaline enzyme mainly present in animals, plants, and microbes. It is usually derived from egg whites and it can damage bacterial cell walls by catalyzing the hydrolysis of 1,4-b-linkages between muramic acid and *N*-acetyl glucosamine in mucopolysaccharides [1,2,3]. Therefore, it is widely used as a cell-disrupting and potent anti-bacterial reagent. Lysozyme is in high demand due to its many favorable characteristics. It is naturally occurring, non-toxic, and easy to digest and absorb [4]. However, most of the lysozymes are extracted from egg white using conventional methods such as salting out, crystallization, ultrafiltration, reverse micelle extraction, ion exchange, etc. [5,6,7]. The above purification methods are complicated, time consuming, not widely applicable, and even some methods may denature lysozyme. Therefore, it is urgent to find a method that is both cost-effective and rapid to separate and purify lysozyme from egg white.

Recently, magnetic nanoparticles have attracted significant interest in a wide range of applications, such as cell separation, enzyme immobilization, and targeted drug administration [8,9,10]. Magnetic nanoparticles commonly contain elements such as iron, nickel, cobalt, and their alloys or oxides of magnetic elements [11,12,13,14]. The preparation of magnetic nanoparticles mainly involves chemical methods, including pyrolysis, co-precipitation, sol-gel, micro emulsion, ultrasonic chemistry, electrochemical deposition, and laser decomposition [15,16,17,18]. Magnetic nanoparticles have many advantages, such as small size, large surface area, low toxicity, biocompatibility, and superparamagnetic properties [19]. Additionally, the motion trajectory of magnetic nanoparticles can be controlled via external magnetic field conditions [20,21]. Therefore, there have been many studies reported in the literatures concerning the immobilization or adsorption of enzymes with ferrite nanoparticle-polymer composites [22,23]. In particular, magnetic particles known as superparamagnetic nanoparticles, with a particle size less than 30 nm, have been shown to possess superparamagnetic properties with strong magnetic responsiveness [24]. Magnetic particles can be rapidly enriched and positioned under the action of an external magnetic field and the magnetic properties of the particles disappear rapidly when the applied magnetic field is removed. Magnetic nanoparticles have unique advantages when applied to protein immobilization and separation. Their unique magnetic properties allow them to separate and enrich their target products directly from complex systems, enabling high purity and high recovery of samples without expensive instruments and equipment. In addition, the separation process after immobilizing enzymes with magnetic nanoparticles is mild and rapid, and they have small bonding and elution power, which maintains the conformational stability and biological activity of the immobilized enzyme [25,26].

Aptamers are single-stranded DNA or RNA sequences, typically 15–40 bases in length, that are selected from a library of synthetic nucleotide combinations by SELEX (systematic evolution of ligands by exponential enrichment) techniques [27]. The aptamer can bind, with high affinity and specificity, to different molecules, including proteins, peptides, drugs, small organic molecules, and cells [28]. Aptamers also have many advantages, such as a short synthesis cycle, low cost, strong specificity, high binding, good stability, and easy modification, which have facilitated their development in many fields of medical diagnosis and treatment and biological analysis [29,30]. The aptamer of the enzyme can be chemically synthesized rapidly at a large scale using simple and low-cost preparation methods. Aptamers are generally developed in vitro using a defined iterative procedure known as systematic evolution of ligands by exponential enrichment (SELEX) [31]. These methods produce unique structures with high affinity that enable strong and stable binding between the aptamer of the enzyme and the target molecule with little effect on enzyme activity [32]. Furthermore, the specificity of the enzyme aptamer makes it possible to specifically recognize enzymes in complex systems [33,34]. Therefore, in this study, lysozyme was separated from egg white using magnetic nanoparticles with a lysozyme aptamer. The material was separated by the action of an external magnetic field, which typically has a high recovery rate and a low cost.

**Scheme 1:**
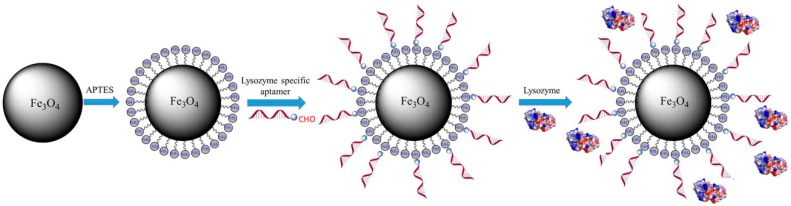
Illustration of the fabrication of Apt-NH_2_-Fe_3_O_4_ NPs for lysozyme immobilization.

Herein, we report a new method for purification of lysozyme that exploits a selective adsorption process by magnetic nanoparticles with a lysozyme sequence aptamer (Scheme 1). Magnetic Fe_3_O_4_ NPs were prepared by a chemical co-precipitation method and were then functionalized with amino groups using 3-aminopropyltriethoxysilane (APTES) to produce NH_2_-Fe_3_O_4_ NPs. The lysozyme aptamer was then attached to the NH_2_-Fe_3_O_4_ NPs using a specific reaction of the aldehyde and amino groups to produce Apt-NH_2_-Fe_3_O_4_ NPs. Different adsorption conditions were tested to determine the optimal conditions for the adsorption of lysozyme in water to the prepared nanomaterials. The variables tested included initial lysozyme concentration, adsorption time, pH, reaction temperature, and ionic strength. The Apt-NH_2_-Fe_3_O_4_ NPs were then used for the separation of lysozyme from crude fresh egg whites.

## 2. Materials and Methods

### 2.1. Materials

Lysozyme was purchased from Sigma (St. Louis, MO, USA). 3-aminopropyltriethoxysilane (APTES), ferrous chloride tetrahydrate (FeCl_2_·4H_2_O), ferric chloride hexahydrate (FeCl_3_·6H_2_O), ammonia (25%), sodium dihydrogen phosphate (NaH_2_PO_4_), disodium hydrogen phosphate (Na_2_HPO_4_), toluene, and anhydrous ethanol were purchased from Beijing Chemical Factory Reagent Company (Beijing, China). A phosphate buffer solution (PBS) was prepared using NaH_2_PO_4_ and Na_2_HPO_4_. The lysozyme-specific aptamer sequences with aldehyde modification (5′-aldehyde-TTTTTTATCAGGGCTAAAGAGTGC-3′) were purchased from the Lianxing Biotechnology Company (Shenyang, China). Ultra-pure water with 18.2 MΩ·cm resistivity was used in all experiments. All chemicals were used as received without any further purification.

### 2.2. Preparation of Nanoparticles

**Synthesis of magnetic Fe_3_O_4_ NPs:** FeCl_2_·4H_2_O (3 g) and FeCl_3_·6H_2_O (9.1 g) were mixed in 200 mL of distilled water that had previously been purged with nitrogen and the solution was stirred for 10 min. After adding 85 mL of ammonia (25%), the solution was stirred at 25 °C for 30 min and then heated to 70 °C with continual stirring for 30 min with nitrogen purge. The reaction products were collected with a magnet after the reaction system had cooled to room temperature and were repeatedly washed with distilled water to remove unreacted material. Finally, the products were freeze-dried and magnetic Fe_3_O_4_ NPs were obtained [19].

**Synthesis of magnetic NH_2_-Fe_3_O_4_ NPs:** The surfaces of Fe_3_O_4_ NPs were coated with APTES using a silanization reaction. After dissolving 4 g of magnetic Fe_3_O_4_ in 50 mL of anhydrous toluene, the solution was homogenized using ultrasonic waves for 15 min and then 22 mmol of APTES was added via syringe. Nitrogen gas was then introduced into the reaction system, which was stirred for 5 h at 25 °C. After the reaction was completed, the reaction products were collected with a magnet, washed repeatedly with ethanol, and dichloromethane and freeze dried. The NH_2_-Fe_3_O_4_ NPs were then obtained.

**Synthesis of magnetic NH_2_-Fe_3_O_4_ NPs with lysozyme aptamers (Apt-NH_2_-Fe_3_O_4_ NPs):** NH_2_-Fe_3_O_4_ NPs (1 g) were dissolved in PBS solution (20 mL, 0.1 M, pH 8.0) containing 10 nM lysozyme aptamer and the mixture was shaken at 200 rpm for 6 h at 35 °C. The reaction products were separated using a magnet, washed repeatedly with distilled water, and freeze dried. The magnetic Apt-NH_2_-Fe_3_O_4_ NPs were then obtained.

### 2.3. Nanoparticle Characterization

The morphology and particle size of the magnetic Fe_3_O_4_ NPs were investigated by transmission electron microscopy (TEM, Tecnai G2F30; FEI, Hillsboro, OR, USA). Samples were prepared for TEM observation by ultrasonic dispersion. Take some powder sample into a centrifuge tube with ethanol and ultrasonically dispersed into a suspension. Then, a few drops of the suspended droplets were taken and dropped on a copper mesh (3 mm in diameter) using a capillary and dried to obtain a powder sample observed by an electron microscope. X-ray diffraction (XRD, Bruker, Karlsruhe, Germany) analysis was performed using the Bruker High-Resolution D8 Advance XRD unit with a Ni-filtered CuK (λ = 1.54 Å). Magnetization performance of the magnetic Apt-NH_2_-Fe_3_O_4_ NPs was characterized by measuring the hysteresis loop (Quantum Design, San Diego, CA, USA). The material was demagnetized before measuring the hysteresis loop and then the sample was magnetized to saturation, the magnetizing current was evenly reduced, and the coordinates of the hysteresis loop corresponding to different magnetization currents were recorded. The magnetic content of the Apt-NH_2_-Fe_3_O_4_ NPs was measured using thermogravimetric analysis (TGA) with the following test conditions: rate of nitrogen purge, 100 mL·min^−1^; rate of temperature rise, 20 °C·min^−1^ and temperature range, 30–900 °C. The functional surface groups of Apt-NH_2_-Fe_3_O_4_ NPs were characterized by Fourier transforms infrared spectroscopy (FTIR, IR Prestige-21; Shimadzu, Kyoto, Japan). Samples were prepared by the KBr tablet method. The KBr was dried at 140 °C for 5 h before the measurement and the ratio of the powder sample to the KBr powder was 1:100. The mixing sample was fully ground and compressed, which was used for infrared measurement and the spectrum range was 400 to 4000 cm^−1^. The corresponding potential values of Apt-NH_2_-Fe_3_O_4_ NPs were calculated by measuring the potential values at different pH conditions using a zeta potential meter (Zetasizer Nano ZS 90; Malvern, UK). The magnetic Apt-NH_2_-Fe_3_O_4_ NPs were dispersed in distilled water by ultrasonic treatment and the pH values of the samples were adjusted with HCl and NaOH solutions. 

### 2.4. Lysozyme Adsorption in an Aqueous Solution

To measure the adsorption equilibrium of magnetic Apt-NH_2_-Fe_3_O_4_ NPs, 10 mg of material and 4 mL of a specific lysozyme buffer solution were mixed in a 5 mL centrifuge tube. Experiments were repeated at different lysozyme concentrations (0.25–5.0 mg·mL^−1^) and at various pH values (4.0–11.0). Adsorption experiments were performed at a stirring speed of 180 rpm for different times. The magnetic nanoparticles were separated from the supernatant using a magnet after the adsorption equilibrium was reached and were then washed with deionized water to remove any lysozyme that was not completely adsorbed onto the materials. The supernatant was analyzed by measuring its absorbance at 280 nm (the molar extinction coefficient of lysozyme at 280 nm is 14,301 M^−1^·cm^−1^), and the amount of lysozyme adsorbed was calculated by determining the lysozyme concentration in the supernatant, according to the following equation:

(1) $$q=\frac{V\left(C_{0}-C_{e}\right)}{m},$$

where *q* is the adsorption capacity of Apt-NH_2_-Fe_3_O_4_ NPs to lysozyme (mg·g^−1^), *C*_0_ is the initial lysozyme concentration in the solution (mg·mL^−1^), *C_e_* is the lysozyme concentration at equilibrium (mg·mL^−1^), *m* is the amount of Apt-NH_2_-Fe_3_O_4_ NPs (g), and *V* is the volume of the lysozyme solution (mL).

### 2.5. The Separation and Purification of Lysozyme from Egg White

Egg white liquid was initially prepared according to a previously published method [14]. The egg whites were separated from fresh eggs and were then mixed with PBS (20 mM, pH 7.2–8.0, volume ratio 1:1). The system was stirred for 6 h in an ice-water bath, the white flocculent precipitates were removed by centrifugation at 10,000 rpm for 30 min at 4 °C and the supernatant was obtained. The supernatant was then used as the lysozyme separation solution and the purified egg white raw material solution. Next, 10 mg of Apt-NH_2_-Fe_3_O_4_ NPs was added to 1 mL of the lysozyme separation solution and the mixture was stirred at 180 rpm for 60 min at 37 °C. The magnetic nanoparticles were then separated by a magnet and washed several times with deionized water to remove unabsorbed substances. The collected magnetic nanoparticle adsorbed with lysozyme was stored at −20 °C for the next elution test and further assayed for the activity of the isolated lysozyme. The supernatant was analyzed by measuring its absorbance at 280 nm and the amount of lysozyme adsorbed was calculated by determining the lysozyme concentration in the supernatant.

### 2.6. Lysozyme Desorption

After separating lysozyme from egg white, the Apt-NH_2_-Fe_3_O_4_ NPs were subjected to a desorption test. The nanoparticles were added to a glycine solution (0.2 M, pH 2.0) and the eluate was collected and analyzed for concentration, purity, and enzyme activity. 

### 2.7. SDS Gel Electrophoresis

The purity of the eluate was measured using sodium dodecyl sulfate-polyacrylamide gel electrophoresis (SDS-PAGE) with a 12% polyacrylamide separation gel and an 8% polyacrylamide concentration gel.

### 2.8. Activity of Lysozyme after Separation

The freeze-dried bacterial strains were first resuscitated and activated. Then, their optical density at 450 nm (OD_450_) was adjusted to 1.3 by diluting the bacterial solution in PBS (0.1 M, pH 7.2–8.0). After dissolving 10 mg of lysozyme in 1 mL of PBS (0.1 M, pH 6.24), it was then diluted to 100 U·mL^−1^ and 0.5 mL of this standard enzyme solution was added to 2.5 mL of the aforementioned bacterial solution. The decrease in absorbance per minute was measured using a microplate reader. Lysozyme activity was calculated using the equation below:

(2) $$\mathrm{Lysozyme}\;\mathrm{activity}\;\left(\frac{U}{\mathrm{mg}}\right)=\frac{\mathrm{Initial}\;\mathrm{absorbance}-\mathrm{Final}\;\mathrm{absorbance}}{0.001\times t\;\left(\min\right)\times \mathrm{Lysozyme}\;\mathrm{quality}\;\left(\mathrm{mg}\right)}.$$

## 3. Results and Discussion

### 3.1. Characterization of the Prepared Nanoparticles

**Figure 1:**
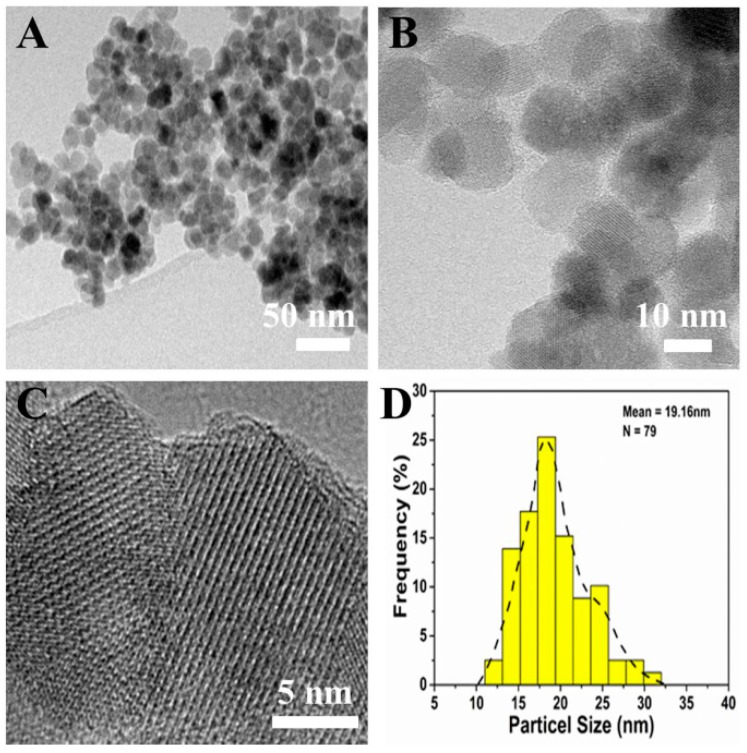
Transmission electron microscopy (TEM) images of Fe_3_O_4_ NPs (**A**–**C**) and size distribution histogram (**D**) of the synthesized Fe_3_O_4_ NPs.

The morphology of the Fe_3_O_4_ NPs was determined by TEM images (Figure 1). TEM images (Figure 1A–C) and size distribution histogram of the synthesized Fe_3_O_4_ NPs (Figure 1D) showed that the magnetic Fe_3_O_4_ nanoparticles had a uniform diameter of about 19 nm and had a spherical shape with a smooth surface. They also showed that the magnetic Fe_3_O_4_ NPs had good dispersion, with basically single layer dispersion. 

**Figure 2:**
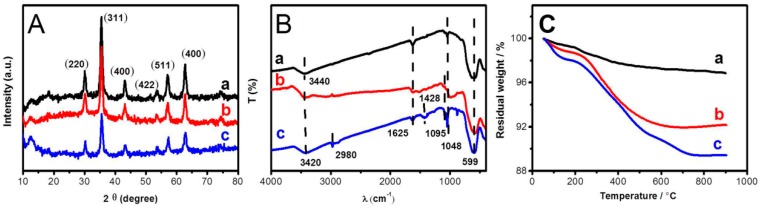
The X-ray diffraction (XRD, **A**), Fourier transform infrared spectroscopy (FTIR, **B**), and thermogravimetric analysis (TGA, **C**) data for Fe_3_O_4_ (a), NH_2_-Fe_3_O_4_ (b), and Apt-NH_2_-Fe_3_O_4_ NPs (c).

As shown in Figure 2A, a, b, and c represent the XRD patterns of the Fe_3_O_4_, NH_2_-Fe_3_O_4_, and Apt-NH_2_-Fe_3_O_4_ NPs, respectively, which have the same crystal face characteristic diffraction peaks of (220), (311), (400), (422), (511), (440) that were present at 30.49, 35.66, 43.25, 53.65, 57.32, 63.02, respectively, which indicated that the Fe_3_O_4_ NPs were synthesized successfully [35]. In addition, this also showed that the diffraction peaks of Fe_3_O_4_ NPs did not change after amino functionalization and grafting of lysozyme aptamers. It was proved that the good crystal structure and good magnetic properties of the nanoparticles were maintained, thus ensuring the magnetic separation of the adsorbent in the experiment. 

As shown in Figure 2B, the surface groups were characterized by FTIR absorption peaks in the range of 400–4000 cm^−1^. The characteristic absorption peak of Fe–O at 599 cm^−1^ was seen, indicating that Fe_3_O_4_ NPs were successfully synthesized. The stretching vibration peaks of the Si–O bond were observed at 1048 cm^−1^ and 1095 cm^−1^, indicating that a Si–O bond existed on the surface of the NH_2_-Fe_3_O_4_ and Apt-NH_2_-Fe_3_O_4_ NPs. The stretching vibration peaks of the C–N bond were observed at 1428 cm^−1^ and a vibration peak associated to imine formation between aldehyde of aptamer and NH_2_ is visible at 1625 cm^−1^**,** indicating that aptamer functionalized on the Apt-NH_2_-Fe_3_O_4_ NPs. The vibration peaks observed at 3440 cm^−1^ on Fe_3_O_4_ NPs may be attributed to –OH. The significant vibration peaks observed at 3420 cm^−1^ and 2980 cm^−1^ represented –NH and –CH_2_, indicating that Fe_3_O_4_ NPs were successfully functionalized with amino groups by APTES, and which demonstrated that silica was successfully encapsulated on the surface of Fe_3_O_4_ NPs. This can prevent agglomeration of the magnetic nanoparticles.

As shown in Figure 2C, a, b, and c represent the TGA curves of the Fe_3_O_4_, NH_2_-Fe_3_O_4_, and Apt-NH_2_-Fe_3_O_4_ NPs, respectively. The process of weight loss of Fe_3_O_4_ NPs could be divided into two relatively independent processes. The first weight loss process took place at 50–200 °C and the weight loss of 0.8%, mainly due to the evaporation of water. The second process occurred at 200–650 °C with a weight loss of 1.9%, mainly due to the decomposition of the surface groups of the Fe_3_O_4_ NPs. The TGA curve of NH_2_-Fe_3_O_4_ NPs could be divided into two relatively independent processes. The first weight loss process took place at 50–210 °C and the weight loss was 1.4%, which was mainly due to the decomposition of the surface groups on the magnetic nanoparticles. The second process took place at 210–650 °C with a weight loss of 6.5%, mainly due to the decomposition of the surface groups of the NH_2_-Fe_3_O_4_ NPs. The TGA curve of the Apt-NH_2_-Fe_3_O_4_ NPs was shown in Figure 2C (c). The process of weight loss could be divided into three relatively independent processes. The first weight loss process occurred at 25–220 °C and involved a loss of 2.26%, which was mainly due to the evaporation of water. The second process occurred at 220–700 °C and involved a weight loss of 10.25%, which was mainly due to the decomposition of the surface groups on the magnetic nanoparticles. The last process occurred at 700–900 °C, but the weight loss was not significant, indicating that the Fe_3_O_4_ content of the magnetic core was about 89.4%. These magnetic nanoparticles contained higher Fe_3_O_4_ content and stronger magnetic force.

**Figure 3:**
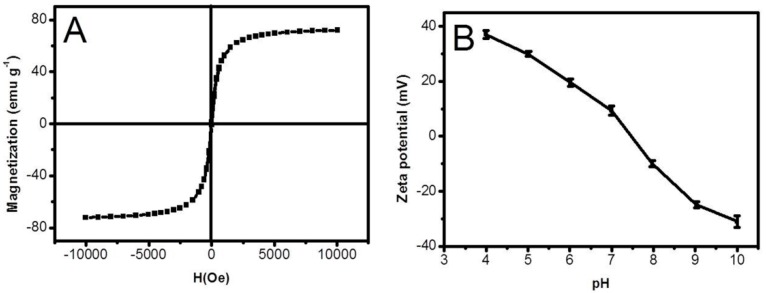
Hysteresis loop (**A**) and zeta potential (**B**) of Apt-NH_2_-Fe_3_O_4_ NPs.

Figure 3A shows the hysteresis loops of the Apt-NH_2_-Fe_3_O_4_ NPs, from which it was observed that the magnetic nanoparticle had a magnetic saturation of 70.9 emu·g^−1^ and the magnetic nanoparticle particles did not exhibit coercivity with an applied magnetic field. As shown in Figure 3A, the hysteresis loop was an S-type loop with a coercive force tending to 0, indicating that the magnetic material had superparamagnetic properties that could prevent the agglomeration of the nanoparticles [19]. The zeta potential of the Apt-NH_2_-Fe_3_O_4_ NPs is shown in Figure 3B. As the magnetic nanoparticles had different groups on their surface, the zeta potentials at different pH values were not the same. The isoelectric point of the magnetic nanoparticles was about 7.6, as shown in Figure 3B. Therefore, when the pH of the solution was greater than 7.6, the magnetic nanoparticles were negatively charged. Lysozyme is positively charged at pH values less than 11.2 (the isoelectric point of lysozyme is approximately 11.2), indicating that magnetic nanoparticles could combine with lysozyme via electrostatic attraction when the pH of the solution was in the range 7.6–11.2.

### 3.2. Adsorption of Lysozyme to Apt-NH_2_-Fe_3_O_4_ Nanoparticles

**Figure 4:**
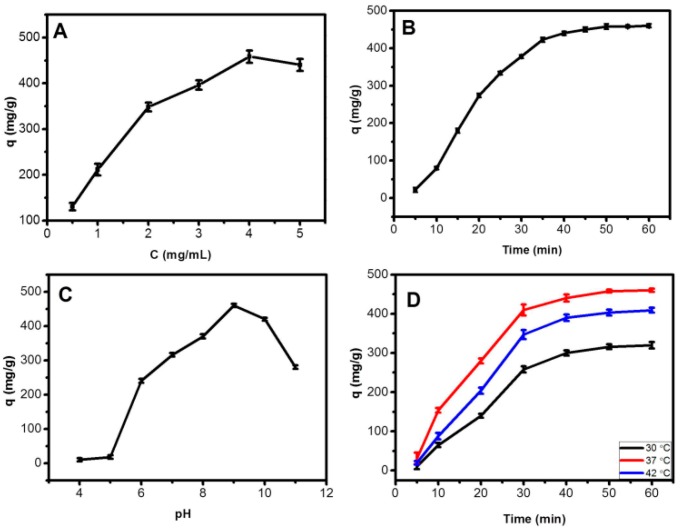
The effect of initial concentration (**A**), adsorption time (**B**), pH (**C**), and reaction temperature (**D**) on the lysozyme immobilization by Apt-NH_2_-Fe_3_O_4_ NPs.

Effect of initial concentration, adsorption time, pH, temperature, and ionic strength: To determine the optimal conditions for lysozyme adsorption onto Apt-NH_2_-Fe_3_O_4_ NPs, the initial lysozyme concentration, absorption time, pH value, temperature, and ionic strength were evaluated. The effect of initial lysozyme concentration was shown in Figure 4A. The amount of lysozyme adsorption increased with the initial lysozyme concentration, until it approached a maximum at a concentration of 4.0 mg·mL^−1^. This concentration maximum could be attributed to the saturation of binding sites on the Apt-NH_2_-Fe_3_O_4_ NPs. The effect of adsorption time on the lysozyme immobilization was studied at a lysozyme concentration of 4.0 mg·mL^−1^ and the temperature was 37 °C. The experimental results showed that the adsorption time reached the maximum at 60 min (Figure 4B). A range of pH values between 4.0 and 11.0 were tested and the optimum pH value for enzyme immobilization by Apt-NH_2_-Fe_3_O_4_ NPs was found to be 9.0 (Figure 4C). Generally, the maximum adsorption of a protein is observed at its isoelectric point, which is pH 11.2 for lysozyme. However, maximum adsorption in this study was found to occur at pH 9.0, which may be due to the positive charge of lysozyme and the negative charge of Apt-NH_2_-Fe_3_O_4_ NPs at this pH [36]. Therefore, the pH-dependent adsorption of lysozyme by Apt-NH_2_-Fe_3_O_4_ NPs indicated that electrostatic attraction was the major force for lysozyme immobilization onto Apt-NH_2_-Fe_3_O_4_ NPs. The effect of temperature on lysozyme adsorption was studied by varying temperatures between 30 and 42 °C (Figure 4D). The optimal temperature was found to be 37 °C. Temperature affected the rate of molecular movement and influenced interactions between proteins. Amino acid residues that are usually buried within the protein may have become exposed on the surface at higher temperatures, resulting in an increase in the binding sites of the protein for the adsorbent. In addition, the NaCl solution was added to the solution to adjust the ionic strength. The NaCl concentration was 0 M and 1 M, respectively. The experimental results are shown in Figure S1. It was known that with the increase of ionic strength, the adsorption capacity of magnetic Apt-NH_2_-Fe_3_O_4_ nanoparticles for lysozyme got worse. Since the ionic strength of the solution was increased, the electrostatic force between the carrier and the lysozyme was reduced and the hydrophobic action was enhanced such that the adsorption was not favorable. Studies have shown that as the salt concentration increases, it would also cause the salting out reaction of the protein and then cause aggregation and precipitation. Therefore, it was concluded that in the adsorption process, 0 M NaCl was added, which was the optimal adsorption reaction condition. Under optimum immobilization conditions, the amount of immobilized lysozyme was 460 ± 3.4 mg·g^−1^ and the activity of the immobilized lysozyme was 16,412 ± 55 U·mg^−1^. This was a higher carrier capacity than those previously reported in the literature, probably due to the specificity of the lysozyme aptamer and the magnetic properties of Fe_3_O_4_ NPs (Table 1) [37,38,39,40]. In addition, the lysozyme immobilization experiment using Fe_3_O_4_ NPs and NH_2_-Fe_3_O_4_ NPs under the optimal conditions were carried out for evaluating this adsorption process. The research showed that this adsorption process is not non-selective adsorption, Fe_3_O_4_ NPs and NH_2_-Fe_3_O_4_ NPs can also achieve lysozyme immobilization, but the immobilization was lower as 218.39 and 211.35 mg·g^−1^, respectively. Therefore, the Apt-NH_2_-Fe_3_O_4_ NPs have higher loading capacity and better performance.

Adsorption isotherms: The adsorption behavior of a lysozyme solution is usually described using the Langmuir and Freundlich isotherm models. The most suitable isotherm model for the data in this study was determined by comparing the correlation coefficients (*r*^2^) of the two models.

The Langmuir curve assumes that the adsorption process is a monolayer, which is applicable to a homogeneous adsorption surface where all adsorption sites have equal adsorbate affinity. It is represented by the following equation [41]:

(3) $$\frac{1}{q_{e}}=\frac{1}{q_{max}}+\frac{1}{q_{max}bC_{e}},$$

The Freundlich isotherm model assumes heterogeneity of adsorption surfaces and is expressed by the following equation [42]:

(4) $$\ln q_{e}=\frac{1}{n}\ln Ce+\ln K_{F},$$

where, *C_e_* is the concentration of lysozyme in the solution (mg·mL^−1^), *q_e_* is the amount adsorbed at the concentration equilibrium state (mg·g^−1^), *q_max_* is the maximum adsorption capacity at equilibrium (mg·g^−1^), and *b* is the adsorption constant.

**Figure 5:**
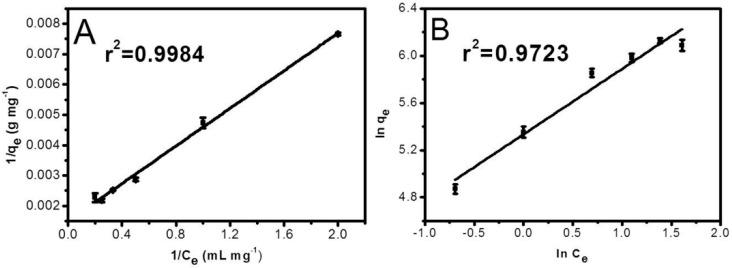
The Langmuir isotherm (**A**) and Freundlich isotherm (**B**) curves for lysozyme adsorption onto Apt-NH_2_-Fe_3_O_4_ NPs.

Figure 5 shows the Langmuir (A) and Freundlich (B) isotherm curves for lysozyme adsorption by Apt-NH_2_-Fe_3_O_4_ NPs. The parameters of Langmuir and Freundlich are shown in the Table 2. The *r*^2^ obtained from the Langmuir model was slightly higher than that obtained from the Freundlich model, indicating that lysozyme adsorption by Apt-NH_2_-Fe_3_O_4_ NPs may be explained using the Langmuir model.

Adsorption kinetics modeling: In order to examine the controlling mechanism of the lysozyme adsorption process based on Apt-NH_2_-Fe_3_O_4_ NPs, this experiment was investigated. According to the experimental conditions optimized in the above experiments, we weighed 10 mg of magnetic Fe_3_O_4_ nanoparticles and 4 mL of 4 mg·mL^−1^ lysozyme solution in a 5 mL centrifuge tube at 37 °C and shook it for 120 min at 180 rpm. The kinetic models can be used in this case assuming that the measured concentrations are equal to the adsorbent surface concentrations [43]. The quasi-first-order kinetic model is one of the most widely used kinetic equations. It is represented by the following equation:

(5) $$\frac{1}{q_{t}}=\left(\frac{k_{1}}{q_{1}}\right)\left(\frac{1}{t}\right)+\frac{1}{q_{1}},$$

where *q_t_* is the adsorption amount at different times (mg·g^−1^), *q*_1_ is the equilibrium adsorption amount obtained by fitting (mg·g^−1^), and *k*_1_ is the first-order reaction rate constant (min^−1^).

The quasi-second-order kinetic model is also commonly used to describe and analyze the adsorption of lysozyme. From this model, it is possible to deduce whether the surface reaction is a rate control step. It is represented by the following equation [44]:

(6) $$\frac{t}{q_{t}}=\frac{1}{k_{2}{q_{2}}^{2}}+\frac{t}{q_{2}},$$

where *q_t_* is the adsorption amount at different times (mg·g^−1^), *q*_2_ is the equilibrium adsorption amount obtained by fitting (mg·g^−1^), *k*_2_ is the second-order reaction rate constant (g·mg^−1^·min^−1^), and *k*_2_*q*_2_^2^ is the initial adsorption rate (mg·g^−1^·min^−1^).

**Figure 6:**
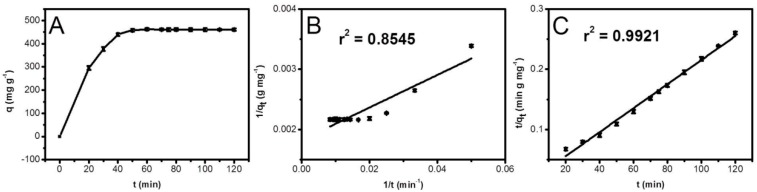
Adsorption kinetics curves (**A**), simulation curves of pseudo-first-order (**B**) and pseudo-second-order (**C**) models of lysozyme onto Apt-NH_2_-Fe_3_O_4_ NPs.

The fitting curves of the quasi-first-order and quasi-second-class models are shown in Figure 6B,C and the fitting curve parameters are listed in Table 3. The *r*^2^ value of the quasi-secondary model (0.9921) was compared with the quasi-first-order model (0.8545) was larger and closer to 1, and the experimental values (460 ± 3.4 mg·g^−1^) of the equilibrium adsorption amount and the calculated values *q*_2_ of the quasi-secondary model agreed well, indicating that the quasi-secondary model is better to predict the adsorption kinetics of lysozyme on Apt-NH_2_-Fe_3_O_4_ NPs.

**Figure 7:**
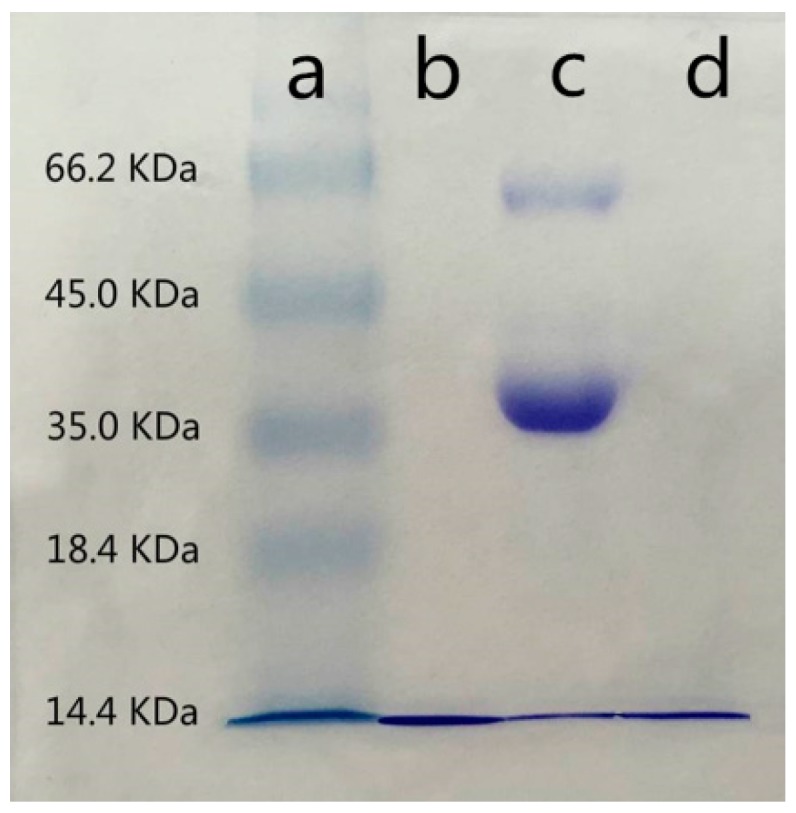
SDS gel electrophoresis of lysozyme separated from whole egg white: (**a**) protein standard markers, (**b**) commercial lysozyme, (**c**) total whole egg white before adsorption, and (**d**) lysozyme eluted from the Apt-NH_2_-Fe_3_O_4_ NPs.

Separation of lysozyme from egg white using Apt-NH_2_-Fe_3_O_4_ NPs: As seen in Figure 7c, there are several main proteins in egg white and a band of lysozyme could be clearly seen in the eluant after the adsorption and desorption by Apt-NH_2_-Fe_3_O_4_ NPs. This indicated that the lysozyme concentration in egg white was high and that the magnetic Apt-NH_2_-Fe_3_O_4_ NPs could separate lysozyme from the egg white (Figure 7d). In addition, the amount of lysozyme separated was 113 ± 4.2 mg·g^−1^, and the activity of the separated lysozyme was 16,370 ± 46 U·mg^−1^. The conventional separation/purification methods for lysozyme are ultra-filtration, precipitation, and chromatography. We present a simple one-step purification of lysozyme from whole chicken egg white samples. The resulting Apt-NH_2_-Fe_3_O_4_ NPs possessed an excellent adsorption and purification of lysozyme. It is easy to recycle and can be reused many times.

### 3.3. Lysozyme Activity Assays

The absorbance value at 450 nm was measured using a microplate reader. Standard enzyme activity was calculated to be 18,400 ± 53 U·mg^−1^ according to the difference in absorbance before and after the reaction. The activity of the separated lysozyme from egg crude was 16,370 ± 46 U·mg^−1^.

## 4. Conclusions

To summarize, magnetic Fe_3_O_4_ NPs were successfully synthesized and were functionalized with amino and lysozyme aptamers to finally obtain Apt-NH_2_-Fe_3_O_4_ NPs for the separation of lysozyme. The resulting Apt-NH_2_-Fe_3_O_4_ NPs possessed an excellent adsorption of lysozyme in water, with a maximum adsorption capacity of 460 ± 3.4 mg·g^−1^ and activity of the immobilized lysozyme was 16,412 ± 55 U·mg^−1^ under the optimal conditions. The optimum initial lysozyme concentration, adsorption time, pH, and reaction temperature were 4 mg·mL^−1^, 60 min, 9.0, and 37 °C, respectively. Furthermore, the lysozyme adsorption process using Apt-NH_2_-Fe_3_O_4_ NPs could be explained using the Langmuir model and a quasi-first-order kinetic model. In addition, Apt-NH_2_-Fe_3_O_4_ NPs could be successfully used to separate lysozyme from egg white with an amount up to 113 ± 4.2 mg·g^−1^, and the activity of the separated lysozyme was 16,370 ± 46 U·mg^−1^. The isolated lysozyme showed high activity and purity. Its purity was demonstrated using SDS-PAGE electrophoresis analysis. The method described here for adsorption and separating enzymes is simple, specific, mild, uses magnetic separation, and has potential in the food field.

## Figures and Tables

**Table 1:** Comparisons of the amount of immobilized lysozyme.

Immobilized Enzyme Carrier	Lysozyme Adsorption Capacity (mg·g^−1^)	References
The hydrophobic affinity ligand l-tryptophan immobilized magnetic poly(glycidyl methacrylate) [m-poly(GMA)] beads	259.6	[37]
Magnetic poly(2-hydroxyethyl methacrylate) mPHEMA beads carrying Cibacron Blue F3GA (mPHEMA/Cibacron Blue F3GA beads)	342	[38]
Macroporous chitosan (CS)/carboxymethylcellulose (CMC) blend membranes beads	240	[39]
Chitosan (CS)/hydroxyapatite (HAP) hybrid membrane	203.9	[40]
Apt-NH_2_-Fe_3_O_4_ NPs	460	This work

**Table 2:** Langmuir and Freundlich parameters for adsorption of lysozyme onto Apt-NH_2_-Fe_3_O_4_ NPs.

Langmuir Adsorption Isotherm			Freundlich Adsorption Isotherm		
*q_e_* (mg·g^−1^)	*b* (mL·mg^−1^)	*r* ^2^	*K_F_*	1/*n*	*r* ^2^
666	2.067	0.9984	0.5546	1.803	0.9723

**Table 3:** Kinetic parameters for the adsorption of lysozyme onto Apt-NH_2_-Fe_3_O_4_ NPs.

Pseudo-First-Order			Pseudo-Second-Order			
*q*_1_ (mg·g^−1^)	*k*_1_ (min^−1^)	*r* ^2^	*q*_2_ (mg·g^−1^)	*k*_2_ (g·mg^−1^·min^−1^)	*k*_2_*q*_2_^2^ (mg·g^−1^·min^−1^)	*r* ^2^
555. 56	15	0.8545	526.32	0.000227	62.89	0.9921

## Supplementary Materials

The following are available online at http://www.mdpi.com/2304-8158/8/2/67/s1, Figure S1: The effect of ionic strength on the lysozyme immobilization using Apt-NH_2_-Fe_3_O_4_ NPs.
