# Supplementary material for: Lysozyme Aptamer-Functionalized Magnetic Nanoparticles for the Purification of Lysozyme from Chicken Egg White
Source: Foods. 2019 Feb 12;8(2):67. doi: 10.3390/foods8020067 (PMC6406557; doi:10.3390/foods8020067)
Supplement: Supplementary file 1 [file foods-08-00067-s001.pdf]

Supplementary Material

Lysozyme Aptamer-Functionalized Magnetic Nanoparticles for the Purification of Lysozyme from Chicken Egg White

Ruiping Luo, Xinrui Zhou, Yan Chen, Sicheng Tuo, Fulin Jiang, Xiaodi Niu, Fengguang Pan *, and Hongsu Wang *

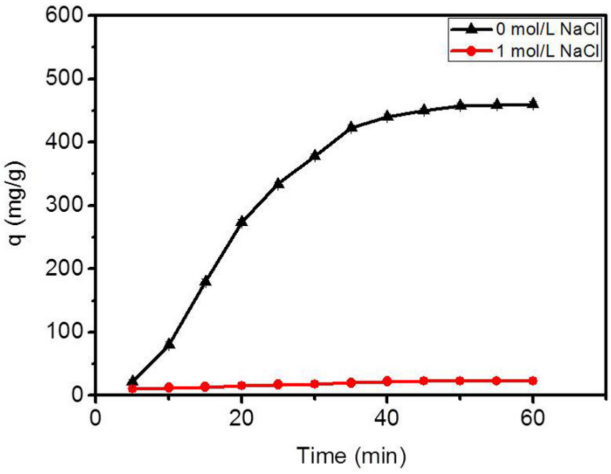

Figure S1. The effect of NaCl on the lysozyme immobilization by Apt-NH_2-Fe_3O_4 NPs
